# The relationship between dietary exposure to persistent organic pollutants from fish consumption and type 2 diabetes among First Nations in Canada

**DOI:** 10.17269/s41997-021-00484-w

**Published:** 2021-06-28

**Authors:** Lesya Marushka, Xuefeng Hu, Malek Batal, Constantine Tikhonov, Tonio Sadik, Harold Schwartz, Amy Ing, Karen Fediuk, Hing Man Chan

**Affiliations:** 1 Environmental Public Health Division, First Nations and Inuit Health Branch, Indigenous Services Canada, Ottawa, ON Canada; 2 Department of Biology, University of Ottawa, 30 Marie Curie, Ottawa, ON K1N 6N5 Canada; 3 Département de Nutrition, Faculté de Médecine, Pavillon Liliane de Stewart, Université de Montréal, C.P. 6128, succ. Centre-Ville, Montréal, QC H3T 1A8 Canada; 4 Centre de recherche en santé publique de l’Université de Montréal et du CIUSS du Centre-sud-de-l’Île-de-Montréal (CReSP), 7101 avenue du Parc, Montréal, QC H3N 1X7 Canada; 5 Assembly of First Nations, 55 Metcalfe Street, Suite 1600, Ottawa, ON K1P 6L5 Canada

**Keywords:** Dichlorodiphenyldichloroethylene (DDE), Polychlorinated biphenyls (PCBs), First Nations, Type 2 diabetes, Fish consumption, Dichlorodiphényldichloroéthylène (DDE), polychlorobiphényles (PCB), Premières Nations, diabète de type 2, consommation de poisson

## Abstract

**Objective:**

We previously examined the associations between dietary dichlorodiphenyldichloroethylene (DDE) and polychlorinated biphenyls (PCBs) intake from fish consumption and type 2 diabetes (T2D) prevalence in Ontario and Manitoba. This study aims to further explore the relationship in a regionally representative sample of First Nations adults living on-reserve across Canada.

**Methods:**

Dietary, health and lifestyle data collected by the cross-sectional First Nations Food, Nutrition and Environment Study (2008–2018) were analyzed. This participatory study included 6091 First Nations adult participants who answered questions on T2D. The consumption of locally caught fish was estimated with a food frequency questionnaire. A total of 551 samples from 96 fish species were collected and analyzed for the presence of DDE and PCBs. The associations between fish and dietary DDE/PCBs intake with self-reported T2D were investigated using multiple logistic regression models adjusted for confounders.

**Results:**

Dietary exposure to DDE (>2.11 ng/kg/bw) and PCBs (>1.47 ng/kg/bw) vs no exposure was positively associated with T2D with ORs of 2.33 (95% CI: 1.24–4.35) for DDE and 1.43 (95% CI: 1.01–3.59) for PCBs. The associations were stronger among females (DDE OR = 3.11 (1.41–6.88); PCBs OR = 1.76 (1.10–3.65)) and older individuals (DDE OR = 2.64 (1.12–6.20); PCBs OR = 1.44 (1.01–3.91)) as compared with males and younger participants. Also, significant dose-response relationships were found for fish consumption in females only.

**Conclusion:**

This study confirms our previous findings that dietary DDE/PCBs exposure may increase the risk of T2D. The effect of DDE/PCBs from fish consumption is driven by geographical differences in DDE/PCBs concentrations in fish and by the amount of fish consumed, and is more prominent in females than in males.

## Résumé

**Objectif:**

Nous avons précédemment examiné les associations entre l’apport alimentaire de dichlorodiphényldichloroéthylène (DDE) et de polychlorobiphényles (PCB) provenant de la consommation de poisson et la prévalence du diabète de type 2 (DT2) en Ontario et au Manitoba. Cette étude vise à explorer davantage la relation dans un échantillon régionalement représentatif d’adultes des Premières Nations vivant dans des réserves partout au Canada.

**Méthode:**

Les données sur l’alimentation, la santé et le mode de vie recueillies par l’Étude transversale sur l’alimentation, la nutrition et l’environnement chez les Premières Nations (2008–2018) ont été analysées. Cette étude participative comprenait 6 091 participants adultes des Premières Nations qui ont répondu à des questions sur le DT2. La consommation de poisson pêché localement a été estimée à l’aide d’un questionnaire de fréquence alimentaire. Au total, 551 échantillons de 96 espèces de poissons ont été prélevés et analysés pour la présence de DDE et de PCB. Les associations entre la consommation de poisson et l’exposition aux DDE/PCB avec le DT2 auto-déclaré ont été étudiées à l’aide de modèles de régression logistique multiples ajustés pour les facteurs de confusion.

**Résultats:**

L’exposition alimentaire au DDE (>2,11 ng/kg/pc) et aux PCB (>1,47 ng/kg/pc) par rapport à l’absence d’exposition était positivement associée au DT2 avec des OR de 2,33 (IC à 95% : 1,24–4,35) pour le DDE et 1,43 (IC à 95% : 1,01–3,59) pour les PCB. Les associations étaient plus fortes chez les femmes (DDE OR = 3,11 (1,41–6,88); PCB OR = 1,76 (1,10–3,65)) et les individus plus âgés (DDE OR = 2,64 (1,12–6,20); PCB OR = 1,44 (1,01–3,91)) par rapport aux hommes et aux participants plus jeunes. De plus, des relations dose-réponse significatives ont été trouvées pour la consommation de poisson chez les femmes seulement.

**Conclusion:**

Cette étude confirme nos conclusions précédentes selon lesquelles l’exposition à travers l’alimentation aux DDE/PCB peut augmenter le risque de DT2. L’effet du DDE/PCB sur la consommation de poisson est lié aux différences géographiques dans les concentrations de DDE/PCB dans le poisson et à la quantité de poisson consommée, et est plus important chez les femmes que chez les hommes.

## Introduction

Type 2 diabetes (T2D) has become increasingly prevalent among Indigenous populations (Acton et al. 2002; Crowshoe et al. 2018; Young et al. 2000). In Canada, First Nations are disproportionally affected by T2D and related complications compared with non-Indigenous population (Crowshoe et al. 2018; Young et al. 2000). Recent data indicate that, overall, 19.2% of First Nations adults (18+) living on-reserve have been diagnosed with diabetes (FNIGC 2018), varying from 10% to 26% across different regions of Canada (Batal et al. 2021a) compared with 7.3% among the general Canadian population aged 12 and older (Statistics Canada 2018). T2D is a multifactorial disease caused by a complex interaction among environmental, socio-economic inequality, lifestyle and genetic factors. Lifestyle factors such as obesity, unhealthy diet and lack of physical activity are well-known risk factors for T2D (Day and Bailey 2011). Historically, the diet of First Nations was based on traditional foods harvested from the local environment and consisted of wild game, fish, plants and berries. This food contributes to both nutrient intake and physical fitness (Kuhnlein et al. 2004). Over the past decades, First Nations have been undergoing rapid lifestyle and dietary transitions, moving from a traditional high-nutrient diet toward store-bought food, which is high in energy, fat and sugar (Kuhnlein et al. 2004). This dietary transition has been concomitant with a sedentary lifestyle, contributing to the high rates of obesity and T2D in the First Nations population (Batal and Decelles 2019; Young et al. 2000; Johnson-Down et al. 2015; Reeds et al. 2016).

Recently, exposure to environmental contaminants has been recognized as a new risk factor for T2D (Lee et al. 2018). Evidence from a number of epidemiological studies showed positive associations between exposure to persistent organic pollutants (POPs), such as dichlorodiphenyldichloroethylene (DDE) and polychlorinated biphenyls (PCBs), and T2D (Singh and Chan 2017; Wang et al. 2008; Wolf et al. 2019).

Recent evidence also suggests a potential link between mercury exposure and T2D (He et al. 2013). For example, high mercury levels (>16 μg/L) were associated with increased fasting glucose levels among Inuit living in Nunavik (Cordier et al. 2020).

POPs are lipophilic contaminants that persist in the environment and thus can be bioaccumulated and biomagnified along the aquatic food chain. This represents a serious concern for First Nations relying on these species for nutrition and for whom consumption of fish is essential to their cultural identity, spiritual health and overall well-being (Kuhnlein and Chan 2000). Indeed, fish and seafood are considered the main pathway of exposure to environmental contaminants, such as DDE, PCBs and mercury. Elevated exposure to POPs and mercury has been observed among First Nations consuming locally harvested fish and other traditional wildlife (Seabert et al. 2014).

On the other hand, fish consumption brings significant nutritional benefits contributing to the intake of high-quality protein, essential omega-3 fatty acids—namely, eicosapentaenoic acid (EPA) and docosahexaenoic acid (DHA)—and other nutrients (Ebbesson et al. 2005; Liaset et al. 2019). EPA-DHA intake has been shown to lower T2D risk by decreasing systemic inflammatory markers and circulating blood lipids and reducing insulin resistance (Fedor and Kelley 2009). Several prospective studies reported protective effects of fish consumption on T2D (Nanri et al. 2011; Villegas et al. 2011). However, systematic reviews and meta-analyses reported contradicting results on the associations among fish, EPA-DHA and T2D (Wu et al. 2012; Zhang et al. 2013; Zheng et al. 2012). The discrepancies between the findings on the associations between fish intake and T2D were explained by geographical differences in fish consumption patterns and POP levels in fish, which were not taken into account in the former studies (Lee and Jacobs 2010).

We previously investigated the associations among fish consumption, dietary EPA-DHA intake, and DDE and PCBs exposure with the prevalence of T2D in First Nations living on reserves in Manitoba and Ontario (Marushka et al. 2018; Marushka et al. 2017a; Marushka et al. 2017b). We found that dietary DDE/PCBs intake was positively associated with the prevalence of T2D, whereas fish (EPA-DHA) consumption showed protective associations with T2D. However, there was a regional difference; results were only significant in Ontario but not in Manitoba (Marushka et al. 2018). The relatively high dietary DDE/PCBs exposure from fish may outweigh protective associations of fish (EPA-DHA) on the prevalence of T2D (Marushka et al. 2018). Furthermore, we found gender differences in the association, with stronger positive associations between DDE/PCBs and T2D among females than among males. The objective of this study is to explore further the relationship between dietary DDE and PCBs intake and the prevalence of T2D among First Nations in Canada using an expanded dataset that is regionally representative of all on-reserve First Nations south of the 60^th^ parallel. We also aim to describe dietary DDE/PCBs exposure patterns at the ecozone level for risk management purposes.

## Methods

### Study population

This study analyzed data collected by the First Nations Food, Nutrition and Environment Study (FNFNES), a 10-year cross-sectional participatory study (2008–2018) (Chan et al. 2021a). The FNFNES was designed to assess total diets, traditional food consumption patterns and food-related exposure to environmental contaminants in the First Nations adult population living on reserves south of the 60^th^ parallel across Canada in full partnership with the Assembly of First Nations and the participating communities. The selection of First Nations communities was performed using an ecozone framework comprised of 11 ecozones to represent the diversity of diets of First Nations. An ecozone is a large geographical region identified based on the distribution patterns of plants, animals, geographical characteristics and climate (ecozones.ca). The sampling was random and proceeded in three stages: the regions, the communities and the households. In each household, one adult self-identifying as a First Nation person living on-reserve and aged 19 years or older was invited to participate in the study. More details on the methodology are described elsewhere (Chan et al. 2019; Chan et al. 2021a). Sampling weights were calibrated for non-response and population change from 2008 to 2017 to obtain representative estimates of the total First Nations population south of the 60^th^ parallel in Canada. A set of 500 bootstrap weights were used to produce proper variance estimation.

Overall, 92 First Nations communities participated in the FNFNES. The participation process is described in Chan et al. (2021a). Information on diabetes was not collected in eight communities during the first year of the survey in British Columbia; therefore, the current study includes participants from a total of 84 First Nations communities across 10 Canadian ecozones (Batal et al. 2021a; Chan et al. 2011). The overall participation rate was 78%. Pregnant and breastfeeding women who reported having unknown diabetes (*n* = 4) were excluded from the analyses to avoid potential misclassification of gestational diabetes. The final sample included 6085 participants aged 19 years and over.

### Ethics

Ethics approvals were obtained from the Ethical Review Boards at Health Canada, the University of Northern British Columbia, the University of Ottawa and the Université de Montréal. In addition, the Assembly of First Nations (AFN) Chiefs-in-Assembly passed resolutions in support of this research. Participation in the study was voluntary. Written consent was obtained from each individual after an oral and written explanation of the project (Chan et al. 2021a).

### Data collection

Dietary, socio-demographic, health and lifestyle data were collected using household interviews. Participants were asked to complete a social/health/lifestyle questionnaire (SHL), which collected information on age, gender, weight and height (measured or self-reported), physical activity, smoking status, years of education, household size, employment status, income source and dieting. The study participants were asked if a health care professional had ever told them that they had diabetes. If respondents answered affirmatively to the question, they were further asked about the type of diabetes they had (type 1, type 2, or unknown) and how many years ago they had been diagnosed. Only those participants who reported being diagnosed with T2D were included in this study. The questions on diabetes were only added in year 2 of the FNFNES, and information was not collected in the Boreal Cordillera. Therefore, results were reported in only 10 of 11 ecozones covered by FNFNES.

A traditional food frequency questionnaire (FFQ) was administrated to collect data on the consumption of locally caught traditional foods, including fish and seafood, during the four seasons in the past year (Batal et al. 2021b). The questionnaire was developed based on a comprehensive list of traditional foods that were representative of each participating community. Also, a 24-h dietary recall was completed to collect information on total diets (e.g., both traditional and store-bought foods over the prior 24 hours) (Chan et al. 2021a). Three-dimensional food and beverage models were used to estimate corresponding intake quantities. Age- and gender-specific portion sizes of each traditional food item were determined from the 24-h recall data (Chan et al. 2019).

Body mass index (BMI) was calculated as weight (in kilograms) divided by the square of height (in metres). BMI was calculated using both measured and/or reported heights and weights. Reported or a combination of reported and measured heights and weights were adjusted based on the measured ones (Chan et al. 2019). Based on BMI, all participants were categorized into three groups: normal weight (BMI <25 kg/m^2^), overweight (BMI 25–29.9 kg/m^2^) and obese (BMI ≥30 kg/m^2^).

Physical activity data were self-reported. The study participants were asked to describe their physical activities as either inactive, somewhat active, moderately active or vigorous. For the purpose of this study, inactive and somewhat active physical activity categories were combined. To control for the potential contribution of mercury intake from fish consumption to T2D, we have included the mercury exposure as a covariate in the regression model.

### Fish sampling for contaminant analyses

Fish samples collected for contaminant analyses were representative of all fish species consumed by members in each community as First Nations communities were asked to identify the most commonly consumed fish species and those that are of the most concern from an environmental perspective. Each fish sample was a composite of tissues from up to 5 different fish samples. The collected fish samples were analyzed for the presence of several environmental contaminants, including DDE, total PCBs and methylmercury (MeHg). More detailed information was published elsewhere (Chan et al. 2019; Chan et al. 2021b). Traditional foods collected in British Columbia and Manitoba were analyzed by Maxxam Analytics in Burnaby, British Columbia, while foods collected in other regions were analyzed by ALS Global in Burlington, Ontario (Chan et al. 2019; Chan et al. 2021b). All samples were homogenized to provide a homogeneous sample for subsequent digestion. If required, a moisture value was determined gravimetrically after drying a portion of the blended sample at 105°C overnight.

### DDE and PCBs

Tissue (6 g) was homogenized in dichloromethane and filtered through anhydrous sodium sulphate. The extract was evaporated to 6 ml, and 5 ml was injected onto the gel permeation chromatography column where a fraction of the eluent was collected, concentrated and solvent exchanged to acetone:hexane (1:1). Further clean-up was performed by eluting this extract through PSA (primary secondary amine) columns. The final extract was concentrated, and solvent exchanged to isooctane. Analysis was performed for DDE and PCBs using GC-MS in selective-ion monitoring mode with an electron ionization source. Spiked standards and blank samples were measured for quality analysis/quality control.

### Methylmercury

Samples were prepared by alkaline digestion. A combination of methanol and potassium hydroxide was used to solubilize MeHg for instrumental analysis. Highly selective and sensitive detection was achieved by cold vapour atomic fluorescence spectrometry after the pyrolytic decomposition of the GC eluent. The diluted extract was buffered to a pH of 4.5–5.0 and treated with sodium tetraethylborate, resulting in the ethylation of oxidized mercury species. These volatile ethylated species (as well as elemental mercury) were stripped from the liquid phase with argon gas, retained on Tenaex traps, desorbed back into the sample stream and separated with a GC column. Each ethylated mercury species was released from the column en masse into the sample stream, thermally oxidized to elemental mercury and then detected by cold vapour atomic fluorescence spectrometry.

### Estimation of fish, DDE, PCBs, MeHg and EPA-DHA intake

Fish intake (grams/day) was estimated by using both the FFQ and 24-h dietary recall data. Specifically, the total number of days over the past year when fish consumption was reported via the FFQ was multiplied by the age- and gender-specific portion size of fish species reported through the 24-h recalls (Marushka et al. 2017a). Total dietary DDE, PCBs and MeHg intakes were estimated for each individual as follows: the amount of DDE and PCBs (in nanograms/gram), and MeHg (in micrograms/gram) in each fish species was multiplied by the total amount (g) of each fish species consumed per day; then, DDE, PCBs and MeHg intake from all fish species eaten per day were summed up and divided by the body weight of each participant (e.g., ng/kg bw/day for DDE, PCBs and μg/kg bw/day for MeHg). Community-specific data on DDE, PCBs and MeHg concentrations in fish species were used to estimate dietary contaminant exposure. If the community-specific concentrations were not available, ecozone-specific data were applied. If neither community nor ecozone-based DDE, PCBs and MeHg concentrations were available for a fish species, regional or national average contaminant data were calculated. Dietary assessments were validated through correlation analyses between MeHg intake from traditional foods estimated with the FFQ and mercury concentrations in hair measured in First Nations participants (Tikhonov et al. 2021). Dietary mercury intake was correlated with mercury levels in hair.

The concentrations of EPA and DHA in fish species were derived from the Canadian Nutrient File and captured cooking method (Health Canada 2015). In this analysis, EPA-DHA intake combines the amount of EPA and DHA consumed from fish and is expressed as g/day.

### Statistical analysis

Descriptive statistics include the calculation of proportions for categorical variables, means with standard deviation (SD) and medians with interquartile range for continuous variables. Student *t* tests, analysis of variance (ANOVA) and chi-square tests were applied to test statistical significance between subgroups. Subgroup-stratified statistics by ecozone (*n *= 10) were calculated to describe fish consumption patterns and dietary DDE and PCBs intake. Fish consumption was divided into four categories: no fish, <10 g/day, 10–20 g/day, and >20 g/day. Dietary DDE and PCBs intake were categorized into three groups according to the thresholds for the effects of daily dietary DDE, and PCBs intake on the prevalence of T2D (e.g., breakpoints for DDE and PCBs) estimated in our previous study and published elsewhere (Marushka et al. 2018):

1. no exposure,
2. <2.11 ng/kg bw (<breakpoint for DDE) and <1.47 ng/kg bw (<breakpoint for PCBs), and
3. ≥2.11 ng/kg bw (>breakpoint for DDE) and ≥1.47 ng/kg bw (>breakpoint for PCBs).

Multiple logistic regression models adjusted for confounding factors were fitted to examine the associations between fish consumption and dietary PCBs and DDE intake categories, individually, with T2D. The following potential confounding factors were considered: age, gender, BMI, physical activity (inactive/sedentary, moderate, and vigorous), smoking status (yes and no), energy intake, dieting status (yes and no), food security status (food secure, moderately food insecure, and severely food insecure), years of education, household size, income source (wage, social assistance, and pension), employment status (yes and no), MeHg and EPA-DHA intake, ecozone (Pacific Maritime, Montane Cordillera, Taiga Plains, Boreal Plains, Prairies, Boreal Shield, Taiga Shield, Hudson Plains, Mixedwood Plains and Atlantic Maritime) and region (British Columbia, Alberta, Saskatchewan, Manitoba, Ontario, Quebec and Labrador, and the Atlantic region which combines Nova Scotia, New Brunswick, Prince Edward Island and Newfoundland).

Control variables were added into the models gradually to see the relative contribution of other risk factors and their influence on the magnitudes of the effect. The final models included the following covariates: age, gender, BMI, energy intake, dieting status, physical activity, smoking status, region, ecozone, years of education, household size, income source and employment status, and estimated dietary intake of PCBs or DDE, MeHg and EPA-DHA.

Multiple logistic regression analyses were developed for the total population and subgroups (age groups (<45 years; ≥45 years) and gender). We chose the age of 45 as the cut-off between the groups since it was close to the median age of the study population across different regions of Canada. Variables that did not fit a normal distribution were normalized using the natural logarithmic function (e.g., DDE, PCBs, MeHg and EPA-DHA). Dietary contaminant levels below the limit of detection (LOD) were imputed with half of LOD of PCBs (0.0003 μg/g), DDE (0.0005 μg/g) and MeHg (0.004 μg/g) to avoid errors in the analysis (Chan et al. 2019).

Results with a *p* value of less than 0.05 were considered statistically significant. STATA statistical software 14.2 (StataCorp, College Station, TX, USA) was used to perform all statistical analyses.

## Results

This study included a total of 6085 First Nations participants with available self-reported data on diabetes. Among them, 4883 (80%) reported never to have been diagnosed with diabetes, 136 reported to have been diagnosed with type 1 diabetes, 855 reported having been diagnosed with T2D, and 211 reported “unknown type” of diabetes. Among those with “unknown type” of diabetes, 203 had characteristics of T2D (being overweight or obese, being age 40 years or older at the onset of diabetes, and having an inactive or sedentary lifestyle) and were categorized as T2D for these analyses. Individuals with type 1 diabetes were categorized as without T2D. Therefore, in total, 1058 participants were categorized as with T2D, and 5027 participants were categorized as without T2D (Table 1). The adjusted prevalence of self-reported T2D in the total First Nations population was 18.8% and was similar in females (18.7%) and males (18.9%). The mean BMI was 30.8 kg/m^2^ and was comparable between females (31.2 kg/m^2^) and males (30.1 kg/m^2^). Individuals with T2D were more likely to be older, to have a higher BMI, to be less physically active, and to have lower educational attainment than those without T2D. Smoking was reported by 53% of the study population and was significantly lower among participants with T2D (45.6% vs. 55.4%). Individuals diagnosed with T2D reported significantly lower energy intake (1800 kcal vs 1998 kcal).

**Table 1:** Descriptive characteristics of FNFNES participants who had data on self-reported T2D (*n *= 6091)

	Without T2D		With T2D		
	Mean/%	95% CI/*n*	Mean/%	95% CI/*n*	*p* value
*n*	81.2	5027	18.8	1058	
Age, years	42.4	41.2–43.5	53.7	52.3–55.0	0.001
Female	67.8	3308	67.8	712	0.98
BMI, kg/m^2^	30.2	29.7–30.7	33.4	32.3–34.5	0.001
Physical activity					0.001
Inactive/sedentary	61.6	3135	73.4	773	
Moderate	27.8	1332	19.7	217	
Vigorous	10.6	562	6.9	66	
Smoking	55.4	2800	45.6	485	0.006
Dieting	9.8	490	12.5	138	0.107
Years of education	10.9	10.6–11.1	10.2	9.8–10.6	0.001
Household size, *n*	4.8	4.6–5	4.8	4.3–5.3	0.89
Employment, any	71.7	3317	67.4	608.0	0.10
Income source					0.001
Wage	57.0	2724	48.1	453	
Social assistance	34.8	1826	29.1	320	
Pension	8.2	478	22.8	284	
Energy intake, kcal	1998	1921–2076	1801	1690–1911	0.008
Fish consumers	65.2	3501	75.4	819	0.001
Fish intake, g/day	13.2	9.6–16.7	18.5	12.0–25.1	0.057
DDE, ng/kg bw/day	0.38	0.25–0.51	0.60	0.35–0.85	0.03
PCBs, ng/kg bw/day	0.76	0.52–1.00	1.63	0.82–2.43	0.02
EPA-DHA, g/day	0.12	0.08–0.16	0.15	0.09–0.21	0.18
MeHg, μg/kg bw/day	0.11	0.07–0.15	0.26	0.10–0.61	0.39

*T2D*, type 2 diabetes; *BMI*, body mass index

The age-standardized prevalence of T2D was 21.3% (based on the 2016 Canadian census data)

Employment, any, either full- or part-time employment

*DDE*, dichlorodiphenyldichloroethylene; *PCBs*, polychlorinated biphenyls; *EPA*, eicosapentaenoic acid; *DHA*, docosahexaenoic acid, *MeHg*, methylmercury

*p* values correspond to *t* tests for continuous variables and chi-square tests for categorical variables

Weighted estimates

Overall, 67.1% of First Nations participants reported consuming traditionally caught fish at least once in the prior year with an average daily intake of 14.9 g/day. Individuals with T2D ate more fish (18.5 g/day) than those without T2D (13.2 g/day). Similarly, dietary DDE and PCBs intake was higher in diabetic (DDE 0.60 ng/kg bw/day; PCBs 1.63 ng/kg bw/day) than in non-diabetic (DDE 0.38 ng/kg bw/day; PCBs 0.76 ng/kg bw/day) individuals.

Table 2 summarizes the characteristics of the study participants by dietary DDE and PCBs exposure. The DDE/PCBs intake was categorized based on the breakpoint values for DDE (2.11 ng/kg bw/day) and PCBs (1.47 ng/kg bw/day) we estimated previously (Marushka et al. 2018). Overall, intake of DDE and PCBs was associated with higher fish consumption, higher EPA-DHA intake and MeHg exposure, higher prevalence of T2D, older age, being male, and smaller household size. The BMI, total energy intake, physical activity, education level, and income sources were not different between the DDE/PCBs exposure groups.

**Table 2:** Characteristics of FNFNES participants by dietary DDE and PCBs exposure categories

	DDE				PCBs			
	No exposure	<2.11 ng/kg bw	≥2.11 ng/kg bw	*p* trend	No exposure	<1.47 ng/kg bw	≥1.47 ng/kg bw	*p* trend
*n*	1769	3971	345		1769	3656	660	
Fish intake, g/day	0	13.8 (11–16.7)	119.1 (99.8–138.4)	0.000	0	14.5 (11.2–17.9)	54.5 (37.2–71.8)	0.000
DDE, ng/kg bw/day	0	0.27 (0.22–0.32)	5.40 (4.34–6.45)	0.000	0	0.27 (0.2–0.33)	2.44 (1.72–3.16)	0.000
PCBs, ng/kg bw/day	0	0.78 (0.56–1)	9.39 (5.01–13.77)	0.000	0	0.22 (0.19–0.26)	7.26 (5.72–8.8)	0.000
EPA-DHA, g/day	0	0.10 (0.08–0.13)	1.31 (1.07–1.54)	0.000	0	0.13 (0.1–0.16)	0.49 (0.29–0.68)	0.000
MeHg, μg/kg bw/day	0	0.11 (0.06–0.17)	1.42 (–0.17–3.01)	0.000	0	0.12 (0.06–0.18)	0.64 (0.07–1.2)	0.000
T2D, %	14.1	20.7	26.6	0.001	14.1	19.7	28.4	0.001
Age, years	42 (40.4–43.6)	45.4 (44.3–46.5)	50.8 (47.7–54)	0.001	42 (40.4–43.6)	45 (43.9–46.1)	49.8 (48.0–51.6)	0.001
Female, %	72.6	66.3	54.1	0.007	72.6	68	52.7	0.003
BMI, kg/m^2^	30.1 (29.5–30.7)	31.3 (30.6–31.9)	29.9 (28–31.9)	0.06	30.1 (29.5–30.7)	31.1 (30.6–31.6)	31.5 (29.9–33.2)	0.038
Energy intake, kcal	1928 (1836–2020)	1970 (1895–2045)	2062 (1878–2245)	0.15	1928 (1836–2020)	1948 (1861–2036)	2120 (1987–2253)	0.02
Physical inactivity	63.1	65.2	49.6	0.73	63.1	64.5	62.2	0.96
Smoking, %	58.4	51.8	42.9	0.001	58.4	51.3	50.4	0.002
Dieting, %	9.1	10.4	17.7	0.04	9.1	10.7	12.0	0.12
Education, years	11 (10.6–11.3)	10.6 (10.3–11)	10.8 (10.2–11.5)	0.30	11 (10.6–11.3)	10.7 (10.4–11)	10.4 (9.2–11.7)	0.30
Household size, *n*	5.1 (4.8–5.4)	4.7 (4.4–5.1)	3.7 (3.3–4.1)	0.001	5.1 (4.8–5.4)	4.7 (4.4–5.1)	4.4 (4.0–4.8)	0.001
Employment, %	68.7	71.6	75.7	0.09	68.7	71.4	74.6	0.23
Income source, %				0.20				0.24
Wage	52.9	56.8	52.6		52.9	56.5	56.4	
Social assistance	39.5	31.2	26.4		39.5	32.1	24.6	
Pension	7.6	12.0	21.0		7.6	11.4	19.0	

Values are mean (95% CI) or percentage (%), weighted estimates

*T2D*, type 2 diabetes; *BMI*, body mass index; *DDE*, dichlorodiphenyldichloroethylene; *PCBs*, polychlorinated biphenyls; *EPA*, eicosapentaenoic acid; *DHA*, docosahexaenoic acid, *MeHg*, methylmercury

Physical inactivity combines self-reported inactive and sedentary lifestyle

Multiple logistic regression analyses of the associations between dietary DDE and PCBs exposure and T2D are presented in Table 3. Model 1 was controlled for age, gender, BMI, physical activity, smoking and region. Model 2 was additionally adjusted for total energy intake, education level, household size, income source and employment status. Model 3 was further controlled for log-transformed DDE or PCBs, respectively, along with dietary MeHg and EPA+DHA intake. For the total population, significant positive associations were found between DDE and PCBs with T2D across all models. The associations were stronger for DDE than for PCBs, and in the models further adjusted for MeHg and EPA-DHA intake. The adjusted odds ratios (ORs) for DDE intake of less than 2.11 ng/kg bw/day was 1.28 (95% CI: 0.98–1.64), and for exposure to ≥2.11 ng/kg bw/day of DDE was 2.33 (95% CI: 1.24–4.35). The corresponding ORs for PCBs intake of <1.47 ng/kg bw/day was 1.30 (95% CI: 0.97–1.75) and the exposure to ≥1.47 of PCBs was 1.43 (95% CI: 1.01–3.59).

**Table 3:** Multiple logistic regression analyses of dietary DDE and PCBs exposure and type 2 diabetes

	DDE			PCBs		
	No exposure	<2.11 ng/kg bw	≥2.11 ng/kg bw	No exposure	<1.47 ng/kg bw	≥1.47 ng/kg bw
	Reference	OR (95% CI)	OR (95% CI)	Reference	OR (95% CI)	OR (95% CI)
Total population						
Model 1	1.0	1.28* (0.98–1.67)	2.14** (1.18–3.85)	1.0	1.29 (0.93–1.81)	1.42* (0.91–1.81)
Model 2	1.0	1.26* (0.97–1.65)	2.27*** (1.27–4.01)	1.0	1.27 (0.91–2.01)	1.40** (1.01–3.50)
Model 3	1.0	1.28* (0.98–1.64)	2.33*** (1.24–4.35)	1.0	1.30 (0.97–1.75)	1.43** (1.01–3.59)
Females						
Model 1	1.0	1.49** (1.07–2.07)	2.25** (1.15–4.40)	1.0	1.49** (1.06–2.12)	1.69** (1.10–2.60)
Model 2	1.0	1.50** (1.06–2.09)	2.29** (1.16–4.51)	1.0	1.43** (1.03–2.00)	1.75** (1.12–2.71)
Model 3	1.0	1.65*** (1.18–2.31)	3.11*** (1.41–6.88)	1.0	1.44** (1.01–2.31)	1.76** (1.10–3.65)
Males						
Model 1	1.0	0.91 (0.76–1.38)	1.59 (0.89–3.92)	1.0	0.89 (0.57–1.39)	1.16 (0.35–3.86)
Model 2	1.0	0.89 (0.73–1.33)	1.77 (0.92–4.36)	1.0	0.86 (0.53–1.39)	1.20 (0.43–3.99)
Model 3	1.0	0.99* (0.69–1.73)	1.89 (0.88–6.81)	1.0	0.89 (0.55–1.42)	1.22 (0.40–5.63)
Aged <45 y						
Model 1	1.0	1.36 (0.85–2.17)	1.80 (0.52–7.76)	1.0	1.36 (0.85–2.17)	1.80 (0.48–7.73)
Model 2	1.0	1.38 (0.84–2.26)	1.82 (0.42–7.90)	1.0	1.37 (0.84–2.25)	1.81 (0.46–7.80)
Model 3	1.0	1.49 (0.80–2.77)	2.12 (0.42–9.77)	1.0	1.49 (0.80–2.77)	2.13 (0.46–9.77)
Aged ≥45 y						
Model 1	1.0	1.22 (0.92–1.59)	2.22** (1.17–4.17)	1.0	1.22 (0.83–1.71)	1.43 (0.88–3.04)
Model 2	1.0	1.18 (0.89–1.54)	2.29*** (1.22–4.26)	1.0	1.19 (0.84–1.68)	1.38* (0.96–3.88)
Model 3	1.0	1.28 (0.76–2.17)	2.64*** (1.12–6.20)	1.0	1.22 (0.89–1.67)	1.44** (1.01–3.91)

^*^p < 0.1, ^**^p < 0.05, ^***^p < 0.01; *OR,* odds ratio

2.11 ng/kg bw/day and 1.47 ng/kg bw/day are breakpoints for dietary DDE and PCBs intake, respectively (see Marushka et al. (2018) for more details)

Model 1: adjusted for age, gender, body mass index, physical activity, smoking, ecozone and region

Model 2: model 1 additionally adjusted for energy intake, dieting status, years of education, household size, income source and employment status

Model 3: model 2 additionally adjusted for log-transformed DDE or PCBs, respectively, as well as MeHg and EPA+DHA

*DDE*, dichlorodiphenyldichloroethylene; *PCBs*, polychlorinated biphenyls; *EPA*, eicosapentaenoic acid; *DHA*, docosahexaenoic acid; *MeHg*, methylmercury

In the gender-based stratified analyses, the positive effects of DDE and PCBs on the prevalence of T2D were observed in females only across all models. In the fully adjusted model, the exposure to ≥2.11 ng/kg bw/day of DDE resulted in about a 3-times increase in the prevalence of T2D (OR = 3.11 (95% CI: 1.41–6.88)) compared with the reference group. The exposure to PCBs at the level of ≥1.47 ng/kg bw/day increased the odds of T2D by about 1.8 times compared with no exposure (OR = 1.76 (95% CI: 1.10–3.65)).

Effect estimates of DDE and PCBs were also examined in the fully adjusted models stratified by age groups. Among individuals aged 19–44 years, the associations between DDE (OR = 2.12 (95% CI: 0.42–9.77)) and PCBs (OR = 2.13 (95% CI: 0.46–9.77)) with T2D were positive but not statistically significant. Among individuals aged 45 years and over, the exposure to DDE at the threshold level of ≥2.11 ng/kg bw/day resulted in a 2.6-fold increase in the prevalence of T2D compared with the reference group (OR = 2.64 (95% CI: 1.12–6.20)). Similarly, the exposure to PCBs at the threshold level of ≥1.47 ng/kg bw/day resulted in a 1.4-times increase in the prevalence of T2D compared with the reference (no exposure) category (OR = 1.44 (95% CI:1.01–3.91)). However, the exposure to lower doses of DDE/PCBs was not statistically significantly associated with T2D among older individuals.

Table 4 presents characteristics of the study population by four fish consumption categories (no fish, <10 g/day, 10–20 g/day, and >20 g/day). Higher fish consumption was associated with older age, being male, higher prevalence of self-reported T2D, dieting and being employed. However, the prevalence of smoking and household size were higher among those who consumed less fish. The BMI, level of physical activity, total energy intake and years of education were comparable among participants across the four fish consumption groups.

**Table 4:** Characteristics of FNFNES participants by fish consumption categories

	No fish	<10 g/day	10–20 g/day	>20 g/day	*p* trend
*n*	1769	2537	628	1151	
Fish intake, g/day	0	3.3 (3.0–3.6)	14.0 (13.5–14.5)	68.6 (60.5–76.8)	0.000
DDE, ng/kg bw/day	0	0.10 (0.08–0.12)	0.43 (0.33–0.52)	2.01 (1.46–2.56)	0.000
PCBs, ng/kg bw/day	0	0.41 (0.31–0.50)	1.52 (0.92–2.11)	3.36 (2.01–4.70)	0.000
EPA-DHA, g/day	0	0.02 (0.019–0.03)	0.10 (0.09–0.12)	0.65 (0.54–0.77)	0.000
MeHg, μg/kg bw/day	0	0.09 (0.01–0.17)	0.20 (0.03–0.38)	0.54 (0.11–0.96)	0.000
T2D, %	14.1	19.2	23.7	24.3	0.002
Age, years	42.0 (40.4–43.6)	44.8 (43.6–45.9)	46.5 (44.5–48.5)	47.8 (45.8–49.7)	0.000
Female, %	72.6	71.2	58.0	56.4	0.000
BMI, kg/m^2^	30.1 (29.5–30.7)	31.1 (30.6–31.6)	31.9 (30.4–33.4)	31.0 (29.9–32.0)	0.12
Energy intake, kcal	1928(1835–2020)	1969(1880–2060)	1925(1835–2015)	2020(1895–2145)	0.18
Physical inactivity, %	63.1	65.1	70.5	58.9	0.37
Smoking, %	58.4	53.3	51.4	46.2	0.001
Dieting, %	9.1	9.2	11.1	14.5	0.002
Years of education	11.0 (10.6–11.3)	10.7 (10.5–11.0)	10.4 (9.8–11.0)	10.6 (9.9–11.3)	0.4
Household size, *n*	5.1 (4.8–5.4)	4.8 (4.4–5.1)	4.4 (3.9–5.0)	4.5 (3.9–5.1)	0.01
Employment, any, %	68.7	70.2	71.2	76.0	0.009
Income source, %					0.28
Wage	52.9	56.4	57.6	56.2	
Social assistance	39.5	32.9	26.8	28.6	
Pension	7.6	10.8	15.6	15.2	

Values are mean (95% CI) or percentage (%), weighted estimates

*T2D*, type 2 diabetes; *BMI*, body mass index; *DDE*, dichlorodiphenyldichloroethylene; *PCBs*, polychlorinated biphenyls; *EPA*, eicosapentaenoic acid; *DHA*, docosahexaenoic acid; *MeHg*, methylmercury

Physical inactivity combines self-reported inactive and sedentary lifestyle

The multiple logistic regression analyses on the association between fish consumption and T2D are presented in Table 5. Model 1 was adjusted for age, gender, BMI and region. Model 2 was further adjusted for physical activity, smoking, energy intake, dieting status, education level, household size, income source and employment status. Analyses for the total population showed dose-response relationships across all fish consumption categories with the prevalence of T2D with OR of 1.46 (95% CI: 0.99–2.15) for 10–20 g of daily fish intake and OR of 1.78 (95% CI: 1.20–2.66) for the intake of over 20 g/day of fish when compared with no fish consumption.

**Table 5:** Multiple logistic regression analyses of fish consumption and type 2 diabetes (*n* = 6091)

	No fish	<10 g/day	10–20 g/day	>20 g/day
	Reference	OR (95% CI)	OR (95% CI)	OR (95% CI)
Total population				
Model 1	1.0	1.16 (0.89–1.52)	1.49** (1.03–2.14)	1.74** (1.21–2.51)
Model 2	1.0	1.15 (0.88–1.51)	1.46* (0.99–2.15)	1.78*** (1.20–2.66)
Females				
Model 1	1.0	1.43** (1.06–1.90)	1.46 (0.78–2.73)	2.12*** (1.38–3.27)
Model 2	1.0	1.36** (1.02–1.81)	1.43 (0.77–2.66)	2.08*** (1.31–3.31)
Males				
Model 1	1.0	0.72 (0.45–1.17)	1.22 (0.65–2.28)	1.12 (0.56–2.21)
Model 2	1.0	0.73 (0.45–1.20)	1.29 (0.65–2.57)	1.25 (0.63–2.48)
Aged <45 y				
Model 1	1.0	1.27 (0.81–1.98)	0.70 (0.30–1.59)	2.61** (1.02–6.62)
Model 2	1.0	1.27 (0.79–2.02)	0.74 (0.31–1.78)	2.65** (1.01–6.96)
Aged ≥45 y				
Model 1	1.0	1.09 (0.81–1.47)	1.83** (1.08–3.09)	1.50** (1.04–2.18)
Model 2	1.0	1.06 (0.79–1.43)	1.78** (1.03–3.11)	1.48** (1.01–2.15)

^*^p < 0.1, ^**^p < 0.05, ^***^p < 0.01; *OR*, odds ratio

Model 1: adjusted for age, gender, body mass index, ecozone and region

Model 2: model 1 additionally adjusted for physical activity, smoking, energy intake, dieting status, years of education, household size, income source and employment status

In stratified by gender analyses, the associations between fish intake and T2D were statistically significant in females but not in males. In females, the fully adjusted OR for the highest (>20 g/day) vs the reference (no fish intake) category was 2.08 (95% CI: 1.31–3.31), whereas in males, the respective OR was 1.25 (95% CI: 0.63–2.48). In the analyses stratified by age groups (<45 years vs ≥45 years), consumption of more than 20 g/day of fish vs no fish intake was positively associated with T2D in both groups, with OR of 2.65 (95% CI: 1.01–6.96) in younger individuals and OR of 1.48 (95% CI: 1.01–2.15) in the older age group.

**Fig. 1:**
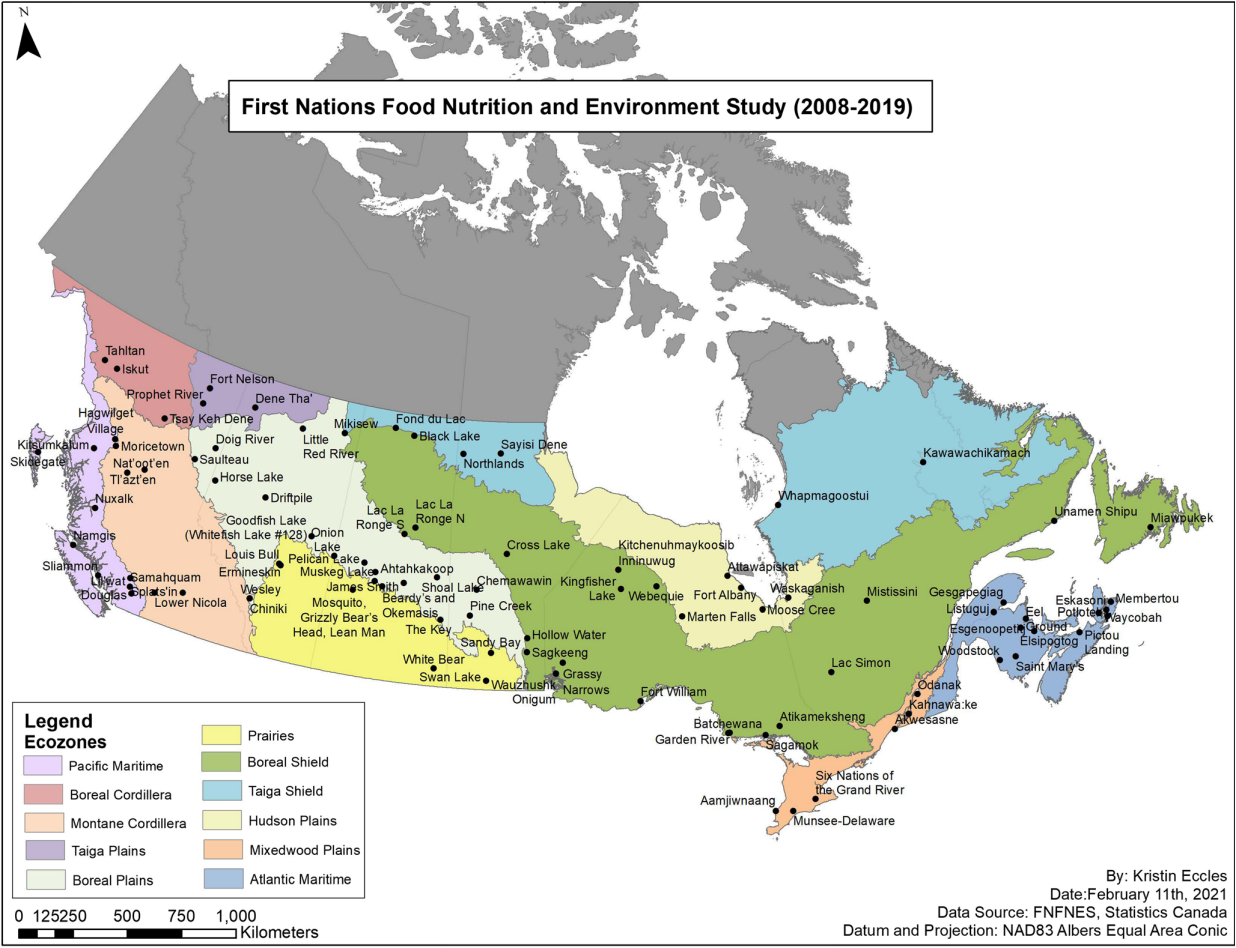
Map of participating First Nations communities across eleven ecozones in Canada

Table 6 in the Appendix summarizes data on the proportion of fish consumers, daily fish consumption, as well as the average dietary DDE/PCBs intake across 10 Canadian ecozones: the Pacific Maritime, Montane Cordillera, Taiga Plains, Boreal Plains, Prairies, Boreal Shield, Taiga Shield, Hudson Plains, Mixedwood Plains and Atlantic Maritime (Fig. 1). Overall, fish consumption was reported by 67.1% of First Nations participants, while ranging from 41.8% to 97.2% across ecozones. The highest fish consumption was reported by First Nations living in the Pacific Maritime ecozone with a mean intake of 76.5 g/day (median 45.4 g/day) followed by 30.0 g/day (median 8.7 g/day) in the Montane Cordillera, 22.6 g/day (median 8.9 g/day) in the Taiga Shield and 22.6 g/day (median 6.6 g/day) in the Boreal Shield. The lowest fish consumption was observed in the Prairies ecozone (3.6 g/day). Dietary DDE and PCBs exposure significantly varied across ecozones. For instance, the highest DDE intake was seen in the Pacific Maritime ecozone with a mean intake of 2.01 ng/kg bw/day (median 1.21 ng/kg bw/day), where almost 30% of participants exceeded the threshold for DDE intake (e.g., 2.11 ng/kg bw/day). On the other hand, the highest dietary exposure to PCBs was observed in the Hudson Plains ecozone with a mean intake of 2.61 ng/kg bw/day and over 23% of respondents with dietary PCBs intake exceeding the breakpoint of 1.47 ng/kg bw/day. The exposure to PCBs was also notably higher in the Mixedwood Plains, Boreal Shield and Taiga Shield ecozones, with 20%, 18.7% and 18% of participants, respectively, exceeding the breakpoint of PCBs (Table 6 in the Appendix).

## Discussion

The findings of this study were similar to our earlier study, i.e., that dietary DDE and PCBs exposure is positively associated with the prevalence of T2D in the overall population. Exposure to the DDE threshold level and over resulted in a 2.3-times increase in the prevalence of T2D, whereas exposure to the PCBs threshold level increased the odds of T2D by 1.4 times compared with the reference group (no exposure). In our earlier findings from Ontario and Manitoba, we found that dietary exposure to ≥2.11 ng/kg bw/day of DDE and ≥1.47 ng/kg bw/day of PCBs was associated with a similar increase of T2D by about 2.3 times for DDE and 1.4 times for PCBs, respectively (Marushka et al. 2018).

Our findings on positive associations between POPs and T2D are in agreement with other epidemiological studies, using cross-sectional design (Everett and Thompson 2012; Lee et al. 2006; Philibert et al. 2009; Singh and Chan 2017; Zuk et al. 2019), and prospective cohort or case-control design (Lee et al. 2011; Raffetti et al. 2018; Tornevi et al. 2019; Wolf et al. 2019; Wu et al. 2013; Zong et al. 2018). However, ORs from the cross-sectional analyses were slightly higher than those from the prospective assessments, which were explained by potential changes in weight and other lifestyle factors among T2D cases (Tornevi et al. 2019).

The risk of diabetes with POPs exposure has also been reported among some Indigenous populations. Among the Mohawk Nation of Akwesasne (located in southern Ontario), positive associations between elevated serum concentrations of PCBs (OR = 3.3) and DDE (OR = 6.4) with diabetes prevalence were observed in a cross-sectional study among adults (≥30 years) (Codru et al. 2007). Positive associations between DDE and PCBs with the prevalence of diabetes and fasting glucose were reported among the Inuit in Canada (Singh and Chan 2017). Positive dose-response associations between POPs and diabetes were found in two Indigenous populations in Quebec (Cordier et al. 2020). A study among Oji-Cree First Nation in Northwestern Ontario examined the associations among self-reported diabetes, fish consumption and serum DDE and PCBs levels. This study reported that exposure to elevated DDE and PCBs was associated with an increased risk of diabetes, whereas consumption of fish, particularly trout and whitefish, showed protective effects (Philibert et al. 2009). Zuk et al. (2019) examined the association between environmental contaminant mixtures and the prevalence of T2D among Eeyou First Nations and found that positive DDT loadings were associated with T2D with a prevalence ratio of 1.27 (95% CI: 1.02–1.60) (Zuk et al. 2019).

It is important to note that the majority of previous epidemiological research investigated blood levels of POPs in relation to T2D, whereas we assessed the effects of dietary POPs intake through the consumption of locally caught fish. However, since exposure to POPs occurs primarily through fish consumption (Fitzgerald et al. 1999; Philibert et al. 2009; Seabert et al. 2014), dietary POP intake from fish is considered a reliable indicator of the exposure. Results of the contaminant exposure assessment of the FNFNES also showed that fish was the main source of DDE and PCBs (Chan et al. 2021b).

We found that stronger positive associations between DDE/PCBs exposure and T2D prevalence were among females and older individuals as compared with males and the younger age group. These findings are consistent with previous studies (Wang et al. 2008; Wolf et al. 2019). Gender differences in the associations between DDE/PCBs exposure and T2D may originate from differences in the body fat composition between females and males (Wahlang 2018). Females tend to have a higher proportion of body fat, which serves as a storage site for lipophilic POPs. However, this needs further investigation. Also, POPs are known as endocrine-disrupting chemicals, which can interfere with the activity of estrogen hormones involved in glucose homeostasis and lipid metabolism (Neel and Sargis 2011).

The underlying mechanisms linking organochlorine pesticides and PCBs with T2D are not fully elucidated. However, several hypotheses have been proposed to explain POP effects on the development of T2D. First, POPs are well-known endocrine-disrupting chemicals that can interfere with estrogen hormones, alter glucose homeostasis and impair cellular insulin action (Lee et al. 2018). Second, low POP exposure can also induce mitochondrial dysfunction and reduce oxidative phosphorylation capacities. Mitochondrial dysfunction is known to play a significant role in chronic low-grade inflammation resulting in ectopic fat accumulation in the liver, muscle and pancreas. Low-grade inflammation, in turn, in adipose tissue promotes the development of insulin resistance. In addition, DL-PCBs can bind to the aryl hydrocarbon receptor and thus inhibit insulin secretion by β-cells, glucose uptake, and functions of insulin in adipose tissue, liver and pancreas (Lee et al. 2018).

Previous studies investigating the relationship between fish consumption and T2D reported that fish and EPA-DHA intake might prevent insulin resistance and glucose tolerance, reduce the levels of inflammatory markers, and improve blood lipid profile (Ebbesson et al. 2005; Liaset et al. 2019; Paquet et al. 2013; Marushka et al. 2017b). Nevertheless, results from systematic review and meta-analyses are inconsistent (Liaset et al. 2019; Telle-Hansen et al. 2019). Growing evidence suggests that the associations between fish consumption and T2D are influenced by the interactions between the levels of toxic chemicals and beneficial nutrients present in fish (Christensen et al. 2016; Lee and Jacobs 2010; Marushka et al. 2018; Wallin et al. 2017). That the positive association between fish as well as dietary DDE/PCBs intake and T2D was observed in the total population and older individuals and was stronger in females suggests that DDE/PCBs concentrations present in fish were the main drivers for the relationship. Furthermore, the associations between DDE/PCBs with T2D were stronger in the models additionally adjusted for EPA-DHA and MeHg intake. This supports our previous findings that elevated levels of DDE/PCBs may diminish the beneficial effects of EPA-DHA on T2D, while relatively high EPA-DHA intake may attenuate the detrimental effects of DDE/PCBs on T2D (Marushka et al. 2018). On the other hand, the co-exposure to MeHg can potentially increase the risk of T2D, particularly among females. The relationship between MeHg exposure and T2D is relatively less studied; more prospective studies are warranted to establish a causal relationship between mercury exposure and the risk of T2D (Roy et al. 2017).

There were differences in the magnitude of the associations between fish and T2D among the younger and older age groups. This may be partially explained by differences in the distribution of males and females in the two age groups. Also, younger individuals consumed less fish (11.6 g/day) than older respondents (19.5 g/day) and consequently have a lower intake of beneficial EPA-DHA. The protective effect of EPA-DHA in older individuals may partly outweigh the detrimental effects of DDE/PCBs.

Previous risk assessment for dietary exposure to PCBs and DDE using the toxicological reference values (TRVs) of 0.13 μg/kg/day for the sum of non-dioxin-like PCBs by Health Canada (Health Canada 2010) and 10 μg/kg/day for DDT established by the FAO/WHO (WHO 2000) found that the health risk was negligible (Chan et al. 2021b). The breakpoints that we used in this study (1.47 ng/kg bw for PCBs and 2.11 ng/kg bw for DDE) were two to three orders of magnitude lower. These may be due to the identification of POPs being endocrine-disrupting chemicals, which can exhibit harmful effects on the hormone regulation at much lower doses than previously reported (Birnbaum 2012). These results suggest that the health risk of PCBs/DDE exposure may have been underestimated. With increasing evidence in the association between POPs exposure and T2D, regulatory agencies may need to review and update the TRVs.

We found notable geographical differences in DDE/PCBs exposure patterns across ecozones, which reflect the distribution and diversity of fish species, the amount of fish consumed, as well as DDE and PCBs concentrations in fish. It would be interesting to perform ecozone-based analyses in order to identify ecozones where fish consumption is positively associated with T2D; however, small sample sizes in some ecozones do not allow the multivariate regression analyses by ecozone.

Detailed results on the concentrations of PCBs and DDE in the species that are the top contributors to the dietary intake by ecozone are reported in Chan et al. (2019, 2021b). Elevated PCBs and DDE are typically found in fish that have high-fat content and/or are at higher trophic levels. In the ecozones where DDE or PCBs intake was higher, such as the Pacific Maritime, Hudson Plains, Mixedwood Plains, Boreal Shield and Taiga Shield, more detailed monitoring programs for PCBs and DDE in fish are needed to ensure that fish consumption does not increase the risk of T2D in the First Nations.

Our study has several strengths. First, the sample is large and regionally representative of all First Nations living on-reserve south of the 60^th^ parallel across different regions and ecozones in Canada. Second, the concentrations of DDE, PCBs and MeHg were measured in locally harvested fish in this study, which allowed accurate estimation of DDE, PCBs and MeHg intake by using community-specific data on contaminant content in fish species. Third, information on EPA-DHA levels in fish, derived from the Canadian Nutrient File (Health Canada 2015), captured cooking methods (baked or broiled, smoked, or raw). Also, our results were adjusted for several dietary and lifestyle risk factors for T2D, along with socio-demographic variables and dietary MeHg exposure. Finally, this study found a strong dose-response relationship among dietary DDE/PCBs, fish intake and T2D. Dietary exposure information is more useful to develop fish consumption advisories.

There are some limitations to this study. First, the cross-sectional design does not allow establishing a causal relationship between dietary DDE/PCBs intake and T2D since potential reverse causality is also possible. Therefore, we performed several sensitivity analyses to examine whether individuals diagnosed with T2D tend to change their diets and lifestyles (described elsewhere: Marushka et al. 2018; Marushka et al. 2017a, b). Specifically, we compared the dietary intake (e.g., energy intake, total fat, saturated fat, protein, carbohydrate, fruit and vegetables, and fish consumption) and lifestyle habits (physical activity, smoking status and dieting status) between participants recently diagnosed with T2D (0–5 years) and individuals who had had T2D for a longer period of time (>5 years). The results showed that there were no statistically significant differences in dietary and lifestyle characteristics between the two groups. In addition, we assessed differences in dietary intake and lifestyle behaviour in participants with and without T2D associated with self-reported dieting status. We found that intakes of fish, dietary n-3 FAs, macronutrients and POP, as well as physical activity, were comparable between the two groups. The results showed that individuals with T2D did not change their diet and lifestyle practices following their diagnosis.

Second, there may be errors associated with our classification of T2D. Generally, there is evidence that First Nations have fewer diagnostic tests and more limited access to healthcare due to various factors such as the remoteness of the First Nations, colonialism and racism (NCCAH 2011; Allen et al. 2020). Therefore, the prevalence of T2D could have been underreported. We compared our estimated T2D prevalence with those reported by the First Nations Regional Health Survey (RHS) Phase 2 (2008–2010) and Phase 3 (2015–2016) (FNIGC 2018, 2012). Overall, the prevalence of diabetes was 20.7% in 2008–2010 and 19.2% in 2015–2016 (FNIGC 2018, 2012), which were in a similar range but slightly higher than our estimates (18.8%). These results suggest that the relationship observed between exposures to POPs as a risk factor for T2D is likely to be underestimated. Finally, there is a potential for misclassification of exposure due to dietary measurement errors.

Since this study could only be conducted when all the data were collected, the results could not be communicated to each participating community individually. We communicated the highlights of the results at a national closing workshop held in Ottawa in November 2019 (Chan et al. 2021a). This closing workshop, the First Nations Food, Nutrition and Environment Forum, was attended by 275 delegates from 140 First Nations, including 60 of the participating First Nations. More dialogue on the implications of the results and discussion on possible interventions with the participating First Nations are needed.

## Conclusion

This study confirmed our previous findings that dietary DDE/PCBs exposure might increase the risk of T2D. The associations between dietary DDE/PCBs intake and T2D prevalence were stronger among females and older individuals as compared with males and younger participants. DDE and PCBs exposure patterns significantly varied across Canadian ecozones.

The findings of this study suggest that the effect of DDE/PCBs from fish consumption is driven by geographical differences in DDE/PCBs concentrations in fish species and by the amount of fish consumed, and is more prominent in females than in males. Fish consumption advisories should be tailored to reflect regional and ecozone differences. The research team is collaborating with First Nation partners and health authorities in knowledge translation and risk management. We advocate that Indigenous health research needs to respect diversity, recognize distinct languages and cultures, and promote individual and community self-determination. We hope that the partnership of this research has built local capacity, and the results will help bring about meaningful positive change for the health of people and the environment.

## Data Availability

Data are owned by each participating community. The Assembly of First Nations is data custodian and any requests will be addressed to AFN through the corresponding author.

## Appendix

**Table 6:** Fish intake and dietary DDE and PCBs exposure by ecozone^*^

Ecozone	Fish (g/day)				DDE (ng/kg/day)				PCBs (ng/kg/day)			
	Mean	Median	IQR	% consumers	Mean	Median	IQR	% >2.11	Mean	Median	IQR	% >1.47
Pacific Maritime	76.5	45.4	20.2–101.2	97.2	2.01	1.21	0.43–2.64	29.8	0.75	0.32	0.09–0.84	14.5
Montane Cordillera	30.0	8.7	2.9–28.9	92.5	1.05	0.31	0.09–1.06	16.1	0.36	0.07	0.01–0.27	5.4
Taiga Plains	13.3	1.6	0–15.2	66.5	0.27	0.02	0–0.21	2.6	0.26	0.004	0–0.09	5.9
Boreal Plains	6.5	1.0	0–5.2	61.7	0.12	0.001	0–0.04	1.2	0.13	0.001	0–0.02	1.3
Prairies	3.6	0.1	0–1.6	41.8	0.05	0.001	0–0.003	0.4	0.10	0.001	0–0.001	1.7
Boreal Shield	22.6	6.6	1.9–21.9	87.1	0.54	0.11	0.01–0.41	6.0	1.25	0.23	0.004–0.98	18.7
Taiga Shield	22.6	8.9	2.7–24.6	92.3	0.90	0.22	0.05–0.90	14.3	0.96	0.26	0.07–0.91	18.0
Hudson Plains	11.2	3.4	1.01–11.7	82.3	0.83	0.06	0.01–0.38	11.5	2.61	0.11	0.01–1.12	23.3
Mixedwood Plains	4.3	0.3	0–3.3	51.0	0.44	0.01	0–0.14	4.1	2.24	0.002	0–0.86	20.0
Atlantic Maritime	10.1	2.7	0–10.4	70.6	0.20	0.04	0–0.20	1.0	0.33	0.06	0–0.30	5.7

*IQR*, interquartile range (e.g., 25th and 75th percentiles); *DDE*, dichlorodiphenyldichloroethylene; *PCBs*, polychlorinated biphenyls

2.11 ng/kg bw/day and 1.47 ng/kg bw/day are breakpoints for dietary DDE and PCBs intake, respectively (see Marushka et al. (2018) for more details)

^*^Since diabetes data were not available for Boreal Cordillera, fish and dietary POP exposure in the Boreal Cordillera ecozone are not presented

Unweighted estimates
